# 可手术的局部晚期非小细胞肺癌的新希望：新辅助免疫治疗

**DOI:** 10.3779/j.issn.1009-3419.2020.01.09

**Published:** 2020-01-20

**Authors:** 舒洋 姚, 毅 张

**Affiliations:** 100053 北京，首都医科大学宣武医院胸科 首都医科大学肺癌诊疗中心 Department of Thoracic Surgery, Xuanwu Hospital, Diagnostic and Treatment Centers of Lung Cancer, Capital Medical University, Beijing 100053, China

**Keywords:** 肺肿瘤, 局部晚期, 新辅助治疗, 免疫治疗, Lung neoplasms, Local advanced stage, Neoadjuvant therapy, Immunotherapy

## Abstract

免疫检查点抑制剂（immune checkpoint inhibitor, ICI）已经成为晚期非小细胞肺癌治疗的重要手段之一。目前ICI在新辅助治疗阶段的使用还非常有限。然而在肿瘤免疫治疗的这个大背景下，新辅助免疫治疗可能有更突出的优势。在这篇综述中，我们会讨论一部分临床前研究以及免疫新辅助治疗临床研究结果，并探讨其可能的作用机制。我们还会特别指出新辅助免疫治疗的潜在危险和一部分需要解决的问题。

## Abstract (en)

Immune checkpoint inhibitor (ICI) has been proven to be a major breakthrough in patients with advanced non-small cell lung cancer. Up to now, neoadjuvant therapy using ICI are rare. However, in the context of cancer immunotherapy, neoadjuvant treatment may offer an extra advantage. In this review, we will disscuss the existing preclinical data and emerging clinical findings in the neoadjuvant setting and its potential mechanism of action. We will also highlight the potential damage and the questions that are required to be answered.

非小细胞肺癌（non-small cell lung cancer, NSCLC）占所有肺癌的85%，患者的预后与初诊时疾病的分期息息相关，Ⅰ期、Ⅱ期和一部分可以接受根治性手术切除的Ⅲa期患者通常被称为“早期”。早期NSCLC 5年总生存（overall survival, OS）率为36%-92%，不可切除的Ⅲ期为13%-36%，而晚期为0%-10%^[1]^。

在过去十几年里，NSCLC领域的主要进展都集中在变更全身系统性治疗来延长晚期患者的生存时间和改善生活质量上。比如，基于组织学类型的化疗时代：培美曲塞联合铂类的化疗和培美曲塞的维持治疗^[2]^。随着分子生物学的发展，酪氨酸激酶抑制剂（tyrosine kinase inhibitor, TKI）开启了有敏感基因突变患者的靶向治疗时代^[3-5]^。再到最近5年出现的免疫检查点抑制剂（immune checkpoint inhibitor, ICI），晚期NSCLC的治疗又进入了一个新的阶段，不论是未经治疗的还是多线治疗后的患者都可能从该治疗中获益^[6, 7]^。

尽管全身系统性治疗的发展非常成功，但是肺癌手术仍然是最有效的治疗策略，也是唯一可能根治疾病的手段。那么新辅助（术前）治疗比如化疗和放疗都被证实有效，到目前为止，ICI在新辅助治疗阶段的使用还非常有限。然而在肿瘤免疫治疗的这个大背景下，新辅助免疫治疗可能有更突出的优势：当原发肿瘤还在的时候，抗原的暴露会极大增强肿瘤特异性T细胞反应的程度和持续时间^[8]^。在这篇综述中，我们会讨论一部分临床前研究以及免疫新辅助治疗临床研究结果，并探讨其可能的作用机制。我们还会特别指出新辅助免疫治疗的潜在危险和一部分需要解决的问题。

## 1 新辅助免疫治疗可能比辅助治疗更有效的证据：临床前研究

一项临床前研究^[9]^在两种自发转移性三阴性乳腺癌小鼠模型中应用不同的免疫联合治疗，观察在原发肿瘤切除前给予的新辅助治疗或者在术后给予的辅助治疗的不同疗效。结果发现不管免疫治疗的类型如何，包括耗竭调节T细胞的抗CD25，或激活效应T细胞和自然杀伤细胞的抗程序性死亡受体1（programmed cell death protein 1, PD-1）单药或抗PD-1联合抗CD137的治疗，新辅助治疗都比辅助治疗在清除微转移方面更有优势，使得更多获得治疗的小鼠达到根治。在分子水平上，新辅助和辅助性ICI治疗导致外周血中肿瘤特异性CD8^+^淋巴细胞明显增多。肿瘤特异性CD8^+^淋巴细胞的高水平预示着更长的生存时间，尤其是那些接受过新辅助ICI治疗小鼠的水平明显高于接受了辅助性ICI治疗的小鼠。可能的机制为：在免疫治疗之后，活化的肿瘤特异性CD8^+^ T细胞数量增多。这些T细胞不仅可以杀死肿瘤的原发灶和转移性淋巴结，还可以进入血液中。此外，肿瘤特异性T细胞应答会导致新的肿瘤抗原释放。这些抗原会由抗原呈递细胞传递给针对不同部位肿瘤（原发灶、转移灶、循环中）的肿瘤特异性T细胞。在原发肿瘤切除之后，发现血液循环中以及微转移部位中，T细胞/肿瘤细胞的比率明显升高，这可能有助于消灭残存的肿瘤组织。在清除肿瘤之后，肿瘤特异性CD8^+^ T细胞数量还会保持稳定。虽然目前还不确定这是否是维持完全缓解状态的必要因素，但是，在新辅助免疫治疗之后，已经观察到在长时间存活小鼠体内的这部分细胞数量保持稳定。后续其他的研究^[10, 11]^也进一步证实在不同的临床前小鼠模型中，新辅助免疫联合治疗的有效性。

2018年美国癌症研究协会年会（American Association for Cancer Research, AACR）上有研究者^[12]^报告了可切除NSCLC的临床前研究，结果发现与术后辅助免疫治疗相比，新辅助免疫检查点抑制剂的联合应用进一步延长了生存时间，减少了远处复发率，诱导了更强烈的抗肿瘤免疫反应。

尽管这些研究还没有应用到临床治疗阶段，而总体来说，这些研究结果反复证实了一个观点：以解除肿瘤介导的免疫抑制和/或激活抗肿瘤免疫为目标的针对免疫系统的治疗在新辅助阶段应用的话，预后更好。相比较而言，不依赖宿主免疫状态的化疗，比如紫杉醇，在新辅助治疗阶段应用的时候就不能把疗效最终转化为生存获益。

## 2 新辅助免疫治疗可能比辅助治疗更有效的证据：临床研究

### 2.1 免疫单药的新辅助临床研究

CA209-159研究：Forde等^[13]^入组未经治疗的Ⅰ期-Ⅲa期可切除NSCLC患者，术前进行了两次纳武单抗（Nivolumab，3 mg/kg，每2周1次），在第4周接受手术治疗。21例最终接受手术治疗的患者中，有20例达到了根治性切除，主要病理缓解率（major pathologic response, MPR）为45%，18个月的无复发生存（recurrent-free survival, RFS）率为73%。PD-L1阳性和阴性的患者对纳武单抗治疗均有反应，病理应答结果与术前肿瘤负荷密切相关。结果显示出纳武单抗可用于术前新辅助治疗，而且副作用可控，没有发生手术延迟。除此以外，也说明了在免疫治疗之后，影像学评估结果与病理学检查结果存在不一致的情况。随后，在2019年美国临床肿瘤学会（American Society of Clinical Oncology, ASCO）年会上，研究者又报告了该研究的长期随访数据，中位随访34.6个月，中位RFS和OS均未达到。20例患者中15例未发生疾病复发且仍存活，2例死亡（1例死于疾病复发）。发生1例长期免疫相关不良事件（皮肤，3级）。长期随访安全性数据进一步证实了纳武单抗用于NSCLC术前新辅助治疗的可行性和安全性。

LCMC3研究：今年ASCO大会^[14]^给出了另一项应用阿特珠单抗新辅助治疗NSCLC患者Ⅱ期研究的中期结果。该研究选取的Ib期/Ⅱ期/Ⅲa期可切除且未接受过治疗的NSCLC患者。计划入组180例，主要研究终点为手术切除时MPR，次要终点是无病生存时间（disease-free survival, DFS）、疾病缓解率和OS等。截止2018年9月5日已入组了101例，患者均应用2次阿特珠单抗（Atezolizumab，1, 200 mg，每3周1次），在第6周进行手术。其中，90例患者接受了手术治疗，84例进行了MPR评估，主要疗效评价人群为77例可评估MPR且表皮生长因子受体（epidermal growth factor receptor, *EGFR*）和间变性淋巴瘤激酶（anaplastic lymphoma kinase, *ALK*）基因突变阴性或未知的患者。另有8例有驱动基因突变（均未取得MPR）。Ⅰ期和Ⅱ期患者全部完成了手术切除。77例主要疗效评价人群中，MPR率为19%（15/77），其中4例取得病理完全缓解（pathologic complete remission, pCR），49%（38/77）患者的病理学缓解率≥50%。按照实体瘤的疗效评价标准（Response Evaluation Criteria in Solid Tumours, RECIST）评估标准，部分缓解（partial remission, PR）为7%（6/90），疾病稳定（stable disease, SD）为89%（80/90）。在安全性方面，观察到2例出现治疗不相关的5级不良事件（adverse event, AE）（术后心源性死亡、因疾病进展死亡），3级及以上AE发生率为6%（6/101）。AE发生率排名前三位的分别是疲劳（20%）、注射反应（11%）、发热（10%）。中期分析超过了既定的无效终点，期待后续结果。

### 2.2 免疫治疗联合化疗的新辅助临床研究

2018年世界肺癌大会上（World Conference on Lung Cancer, WCLC）西班牙研究者报道了NADIM试验的初步结果^[15]^，用4个字来概况就是史无前例。该研究为一项Ⅱ期、单臂、开放的多中心研究，入组局部晚期可切除的Ⅲa N2期NSCLC患者。患者接受3周期紫杉醇+卡铂联合纳武单抗治疗后在第3或4周接受手术治疗，后序贯纳武单抗240 mg *iv* *q2w*，4个月和纳武单抗480 mg *iv* *q4w*，8个月，术后治疗共满1年。研究计划招募46例患者。主要研究终点为24个月DFS率。已经入组了30例患者，其中13例已完成手术。化疗联合免疫治疗的耐受性良好，没有患者治疗拖延。没有患者因为疾病进展（progressive disease, PD）或毒性在术前退出研究。总体临床缓解率评估中，完全缓解（complete remission, CR）为5%，PR为65%。术后病理缓解率评估中，9例（69.2%, 95%CI: 38.6-90.9%）患者取得pCR，2例患者取得MPR。总有效率（overall response rate, ORR）为84.6%（95%CI: 54.6-98.1%）。虽然由于试验开展较晚，试验患者的DFS、OS等指标还没成熟，但NADIM试验80%的病理学显著缓解率，已经是化疗时代最好数据的3倍。

### 2.3 免疫治疗联合免疫治疗的新辅助临床研究

在单药治疗和联合化疗之外，免疫治疗联合免疫治疗也是一种思路。NEOSTAR试验就对比了纳武单抗与纳武单抗联合伊匹单抗作为新辅助治疗，治疗可手术切除的NSCLC患者。两组患者1:1随机分组，每组计划入22例，一组在第1天、第15天、第29天使用纳武单抗（3 mg/kg, *iv*）另一组为纳武单抗联合伊匹单抗（1 mg/kg，*iv*，第1天），之后接受手术治疗。截止2018年9月6日，33例患者被随机，单药组17例，联合组16例，30例完成了新辅助治疗，26例完成了手术。总MPR率为26%（8/31），单药组为25%，联合组为27%。共有5例pCR（单药组2例，联合组3例）。影像学评估ORR为19%。手术并发症包括1例支气管胸膜瘘。治疗相关的不良事件包括1例5级AE死亡（单药组，糖皮质激素治疗肺炎继发的支气管胸膜瘘），其他均为2级-3级AEs。探索性研究结果发现与单药组相比，联合组能诱导不同的T细胞亚群出现更多的增殖，这可能导致了不同的抗肿瘤免疫应答机制。

表 1列出了其他还在进行中的针对可手术切除的NSCLC新辅助免疫治疗相关的临床研究。全球正在开展的4项国际多中心Ⅲ期临床研究均以免疫治疗联合化疗做为新辅助主要方案。CheckMate816研究已完成入组，期待明年能够看到更多大样本量的Ⅲ期研究数据，丰富免疫联合化疗在新辅助研究阶段的数据，并进一步明确其在该人群中的应用价值和安全性。

**1:** NSCLC中正在进行的新辅助ICI试验 Ongoing neoadjuvant ICI trials in NSCLC

NSCLC ClinicalTrials.gov identifier	Study name	Phase of the clinical trial	Trial status	Estimated enrollment
NCT03237377	Neoadjuvant immunoradiation for resectable NSCLC	Phase Ⅱ	Recruiting	32
NCT03197467	Neoadjuvant anti PD-1 immunotherapy in resectable NSCLC (NEOMUN)	Phase Ⅱ	Recruiting	30
NCTO3081689	Neo-adjuvant immunotherapy with nivolumab for NSCLC patients	Phase Ⅱ	Active, not recruiting	46
NCTO2994576	Atezolizurnab as induction therapy in NSCLC (PRINCEPS)	Phase Ⅱ	Recruiting	60
NCTO2818920	Neoadjuvant pembrolizumab (TOP 1501)	Phase Ⅱ	Recruiting	32
NCTO2927301	A study of atezolizumab as neoadjuvant and adjuvant therapy in resectable NSCLC-lung cancer mutation consortium (LCMC3)	Phase Ⅱ	Recruiting	180
NCTO2572843	Anti-PD-L1 in stage IIIa (N2) NSCLC	Phase Ⅱ	Recruiting	68
NCTO2716038	Neoadjuvant MPDL3280A, nab-paclitaxel and carboplatin (MAC) in NSCLC	Phase Ⅱ	Recruiting	30
NCTO3158129	Study of induction checkpoint blockade for untreated stage Ⅰ-Ⅲa NSCLC amenable for surgical resection	Phase Ⅱ	Recruiting	66
NCT03732664	Neoadjuvant nivolumab in resectable NSCLC	Phase Ⅰ	Recruiting	40
NSCLC: non-small cell lung cancer; ICI: immune checkpoint inhibitor; PD-1: programmed cell death protein 1.				

## 3 新辅助免疫治疗的目前存在的挑战

### 3.1 潜在的风险

虽然新辅助免疫治疗前景光明，而ICI诱导的免疫相关AE，尤其是与其他药物联合使用时，可能会影响根治性手术的进行和最终的效果。这些免疫相关AE可以用免疫调节药物如糖皮质激素进行治疗，在一些患者中还会应用英夫利西单抗或其他免疫抑制药物^[16]^。目前尚不知使用免疫抑制药物治疗免疫相关AE是否会影响长期的RFS。但是从已经进行的NSCLC新辅助免疫治疗的相关研究中，没有患者因免疫相关AE而推迟既定手术的时间点，这说明了新辅助治疗的可行性。

### 3.2 尚未解答的问题

现在还有很多关于新辅助免疫治疗尚无法回答的问题。最关键的问题就是需要随机对照的Ⅲ期临床研究来证明新辅助免疫治疗与辅助免疫治疗相比，能更进一步改善生存。

其他的问题还包括：①手术与新辅助免疫治疗的时机问题。临床前在小鼠模型中评估新辅助免疫治疗的研究中，小鼠两次抗体治疗后接受手术，每次治疗间隔两天。结果，发现治疗时间的变化--推迟或缩短新辅助治疗后手术的时间能明显影响总生存^[17]^。该研究中发现在新辅助治疗之后延长肿瘤抗原的暴露时间会导致T细胞由活化状态重新回到功能障碍的状态。确定最佳的新辅助免疫治疗后的手术时机非常具有挑战性。在确定手术时间之前，更重要的是弄明白在哪个时间点T细胞活化后的效应最强。现在，国际新辅助黑色素瘤协会建议临床上采用新辅助治疗时间为6周-8周。这基于各个研究的结果以及为了防止没有疗效患者的病情进展。②如何更好的监测新辅助免疫治疗的效果。传统的做法是通过影像学检查结果，根据世界卫生组织的标准或RECIST标准判断肿瘤负荷的变化来进行治疗效果的临床评估。而在新辅助免疫治疗中，通过传统的评估标准可能低估病理有效率（pCR或MPR），而pCR和MPR是RFS的最佳预测因子。免疫治疗后病理学特征性的改变包括：淋巴细胞浸润（淋巴细胞、浆细胞、淋巴样聚集、巨噬细胞）同时伴有瘢痕愈合样特点（纤维细胞增生和新生血管的形成）。除此以外，我们还需要更多的生物标志物来进行疗效的评估。Forde研究^[13]^中发现无论是在肿瘤组织中还是在外周血中，新生抗原特异性T细胞数量明显上升，因此认为肿瘤突变负荷（tumor mutational burden, TMB）可能作为疗效的预测因子。然而，与之相悖的是今年WCLC上公布了2项晚期肺癌的研究结果^[18, 19]^，发现TMB与免疫治疗的疗效没有相关性。一项Ⅲ期黑色素瘤的研究中发现循环肿瘤细胞DNA（circulating tumor DNA, ctDNA）可能是独立生存预测因子^[20]^。ctDNA也许是另一个有希望的生物标志物。一项Ⅱ期的NSCLC新辅助研究发现了免疫治疗能导致CD4^+^和CD8^+^细胞明显激活^[21]^。CA209-159试验的最新结果显示ctDNA清除以及外周血T细胞增殖可能是疗效预测和监测复发的潜在预测因子。然而，ctDNA和外周血T细胞与MPR甚至OS或DFS是否相关尚不明确。此外，血液收集过程、收集管类型、抗凝剂、血样储存条件、分离血浆的离心速度和血浆储存条件均是临床ctDNA标准化的限制因素。因此，下一步可以寻找能反映肿瘤-免疫系统相互作用和免疫系统-宿主相互作用的生物学标志物，这样有助于临床医生明确哪些患者会从新辅助免疫治疗中获益最多。③哪种新辅助免疫治疗更好？根据前述研究结果，在NSCLC新辅助治疗阶段，两种治疗方案比如化疗联合ICI的疗效数据更好，我们期待头对头的单药与联合治疗的结果，以及观察联合用药的耐受性问题。

## 4 结语

综上所述，根据临床前研究结果以及已经报道的相关临床研究的结果，新辅助免疫治疗有可能成为高复发风险早期NSCLC患者治疗的标准治疗。虽然在ICI新辅助治疗后手术时机的选择，疗效的评价标准，免疫相关的AEs的处理以及治疗方案的最优化方面还需要更多的工作，我们满怀信心期待着未来更多的临床试验带来更丰富的结果，解决这些问题。
